# Correlating the Structure and Gene Silencing Activity of Oligonucleotide-Loaded Lipid Nanoparticles Using Small-Angle X-ray Scattering

**DOI:** 10.1021/acsnano.3c01186

**Published:** 2023-06-06

**Authors:** Michal Hammel, Yuchen Fan, Apoorva Sarode, Amy E. Byrnes, Nanzhi Zang, Ponien Kou, Karthik Nagapudi, Dennis Leung, Casper C. Hoogenraad, Tao Chen, Chun-Wan Yen, Greg L. Hura

**Affiliations:** † Molecular Biophysics and Integrated Bioimaging Division, Lawrence Berkeley National Lab, Berkeley, California 94020, United States; ‡ Small Molecule Pharmaceutical Sciences, Genentech Inc., 1 DNA Way, South San Francisco, California 94080, United States; § Department of Neuroscience, Genentech, Inc., South San Francisco, California 94080, United States; ∥ Chemistry and Biochemistry Department, University of California Santa Cruz, Santa Cruz, California 95064, United States

**Keywords:** lipid nanoparticle, small-angle X-ray scattering, cryogenic electron microscopy, high-throughput screening, PEG-lipid, structure−activity relationship

## Abstract

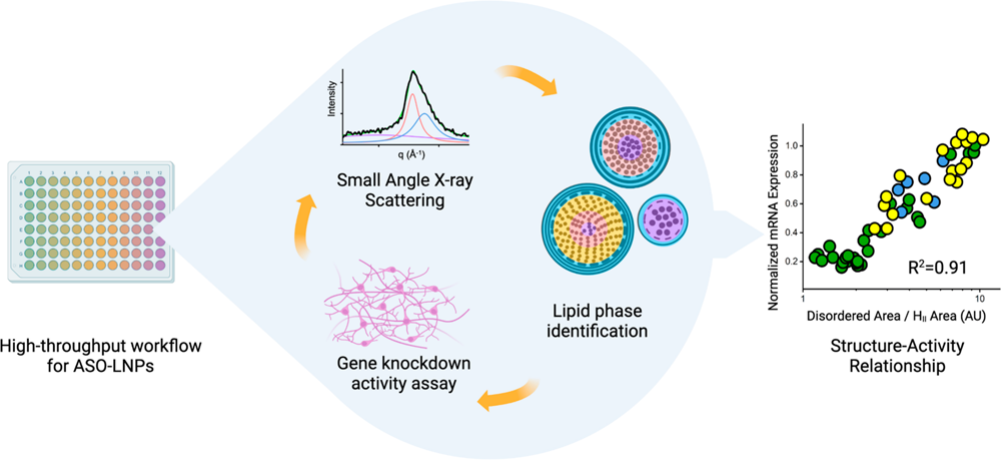

With three FDA-approved products, lipid nanoparticles (LNPs) are under intensive development for delivering wide-ranging nucleic acid therapeutics. A significant challenge for LNP development is insufficient understanding of structure–activity relationship (SAR). Small changes in chemical composition and process parameters can affect LNP structure, significantly impacting performance *in vitro* and *in vivo*. The choice of polyethylene glycol lipid (PEG-lipid), one of the essential lipids for LNP, has been proven to govern particle size. Here we find that PEG-lipids can further modify the core organization of antisense oligonucleotide (ASO)-loaded LNPs to govern its gene silencing activity. Furthermore, we also have found that the extent of compartmentalization, measured by the ratio of disordered vs ordered inverted hexagonal phases within an ASO-lipid core, is predictive of *in vitro* gene silencing. In this work, we propose that a lower ratio of disordered/ordered core phases correlates with stronger gene knockdown efficacy. To establish these findings, we developed a seamless high-throughput screening approach that integrated an automated LNP formulation system with structural analysis by small-angle X-ray scattering (SAXS) and *in vitro TMEM106b* mRNA knockdown assessment. We applied this approach to screen 54 ASO-LNP formulations while varying the type and concentration of PEG-lipids. Representative formulations with diverse SAXS profiles were further visualized using cryogenic electron microscopy (cryo-EM) to help structural elucidation. The proposed SAR was built by combining this structural analysis with *in vitro* data. Our integrated methods, analysis, and resulting findings on PEG-lipid can be applied to rapidly optimize other LNP formulations in a complex design space.

## Introduction

Oligonucleotides, modified chemically for stability and delivery, have shown promise as therapeutics for the treatment of numerous genetic and acquired disorders.^1^ The directed delivery of designed oligonucleotides can differentially regulate pathological target function by promoting gene silencing, activation, or alternative splicing mechanisms. To date, 16 oligonucleotide therapeutics have been approved by the United States Food and Drug Administration (FDA) and the European Medicines Agency (EMA).^2,3^ These successes have provided an impetus to explore oligonucleotide therapeutics more broadly.^2,4,5^ While exogenous nucleic acid administration has proven to be effective in the clinic, inefficient delivery to target tissues remains a significant obstacle to widespread use.^1^ Oligonucleotides are susceptible to enzymatic degradation, can adversely activate the immune system, and have unfavorable physicochemical properties that can prevent cellular internalization.^6^ As a result, safe and effective delivery of these therapeutics often necessitates the use of delivery vehicles such as lipid nanoparticles (LNPs).

Recently, LNP-mediated delivery was utilized in two mRNA vaccines in response to the SARS-CoV-2 (COVID-19) pandemic.^6,7^ LNP delivery systems package the labile oligonucleotides into a primary lipid matrix and aid in transporting the cargo safely across the cell membrane for improved therapeutic efficacy.^8^ Variation in LNP composition stems from mixing different proportions of the four major components: ionizable lipids, helper phospholipids, cholesterol, and polyethylene glycol-lipids (PEG-lipids). Cationic ionizable lipids promote the encapsulation of negatively charged nucleic acids during LNP assembly and aid in cytosolic cargo delivery at the endosomal pH of 5.5–6.5.^9^ While helper phospholipids and cholesterol promote membrane fusion and enhance the endosomal escape of the encapsulated cargo,^10−12^ PEG-lipids have numerous effects governing the LNP properties, including particle size, zeta potential, and stability profile and may determine blood circulation time and clearance rate of LNPs upon systemic administration. The choice of each lipid component and their relative composition ratios in an LNP substantially influence the physicochemical properties as well as its structures.^13,14^

The structural changes of LNP have been observed with varying lipid components,^2,15−19^ N/P ratios (molar ratio of tertiary amines in ionizable lipid to phosphonothioates in oligonucleotide),^20,21^ buffer ionic strength and pH,^20,22^ as well as methods of lipid mixing and LNP preparation.^17^ Cryogenic electron microscopy (cryo-EM) and small-angle X-ray scattering (SAXS) have been used to build models for structural elucidation of LNPs.^15,18,20,21,23−25^ In the previous models, the LNP structures have been described as lipid monolayers enveloping a densely packed core,^15,18,20,21,23−26^ as bilayer structures with a disordered core,^20^ or as multilamellar particles.^14,22^ The LNP core is frequently reported as an inverse hexagonal lipid phase (H_II_) packing the nucleic acid^21,23,24^ or as a core filling with the excess ionizable lipid without interacting with nucleic acid and adopting an amorphous oil phase.^20^ The internal structure of LNPs has also been reported to affect their *in vivo* delivery efficiency and uptake by target cells.^27,28^ Particularly, LNPs with ordered internal morphologies have been reported to enhance intracellular mRNA/antisense oligonucleotide (ASO) delivery as compared to disordered core structures.^15−18^ While these previously described phases provide a framework for understanding LNP structure, their correlation with cellular activity remains unclear with limited study sets currently available.

Commonly, the nucleotide-LNPs are prepared by the microfluidic method^29^ or thin film hydration method.^30,31^ Recently, we successfully developed a high-throughput screening (HTS) workflow for automated, multiplexed preparation of LNP libraries with significant time and material savings.^14^ Using a robotic liquid handler for solvent injection in a 96-well plate format, our workflow can generate hundreds of LNP formulations in a few hours. We further utilized a high-throughput *in vitro* efficacy assay to elucidate the relationship between various LNP compositions and the resulting cellular efficacy.^17^ An existing knowledge gap, and one shared with most formulation campaigns, is the LNP structural contribution underlying optimal oligonucleotide delivery.

While the HTS workflow expanded preparation throughput, structural characterization of the numerous LNP formulations posed a significant bottleneck in advancing our fundamental knowledge. Cryo-EM is most commonly used for the structure elucidation of LNPs. While the high resolution provides information on the shape, size, and internal structure of LNPs, the intricate sample preparation and imaging procedure make it unsuitable for high-throughput screening of large LNP libraries. In contrast, SAXS measured directly in formulation solution provides the structural analysis without complicated sample manipulation. Furthermore, Hura *etal*. developed a high-throughput SAXS (HT-SAXS) methodology enabling a rapid collection of SAXS measurements using minimal sample volumes (30 μL) in a microplate format.^32^ The throughput of HT-SAXS matches the capacity of our HTS generation of LNP formulations and *in vitro* evaluation.

In this study, we integrated our automated formulation process with HT-SAXS to systematically investigate how LNP structural properties influence *in vitro TMEM106b* knockdown efficacy in primary murine cortical neurons. TMEM106b is a lysosomal protein that has been implicated as a risk modifier for frontotemporal lobar degeneration (FTLD)-TDP.^33,34^ Furthermore, the C-terminal fragment has been shown to form amyloid fibrils in various neurodegenerative diseases.^35,36^ We collected SAXS data on a large formulation library of *TMEM106b*-targeting ASO-loaded LNPs (ASO-LNPs) and identified formulations that generated diverse results for cryo-EM analysis. By associating ASO-LNP SAXS signals to their respective cryo-EM features, we identified critical LNP structural parameters that can be analyzed directly from SAXS data. This understanding was then applied to the full library of 54 LNP formulations and their *in vitro* efficacy data sets. We show that specific ASO-LNP morphology, tuned by PEG-lipid parameters, quantitatively predicts *in vitro* efficacy in murine cortical neurons. Our reported methodologies can be applied generally to build a structure–activity relationship (SAR) for LNPs in a high-throughput setup and help identify the chemical composition leading to optimal LNP structure for maximized efficacy.

## Results and Discussion

### Assignment of SAXS Features to LNP Internal Structure Aided by Cryo-EM

**Figure 1:**
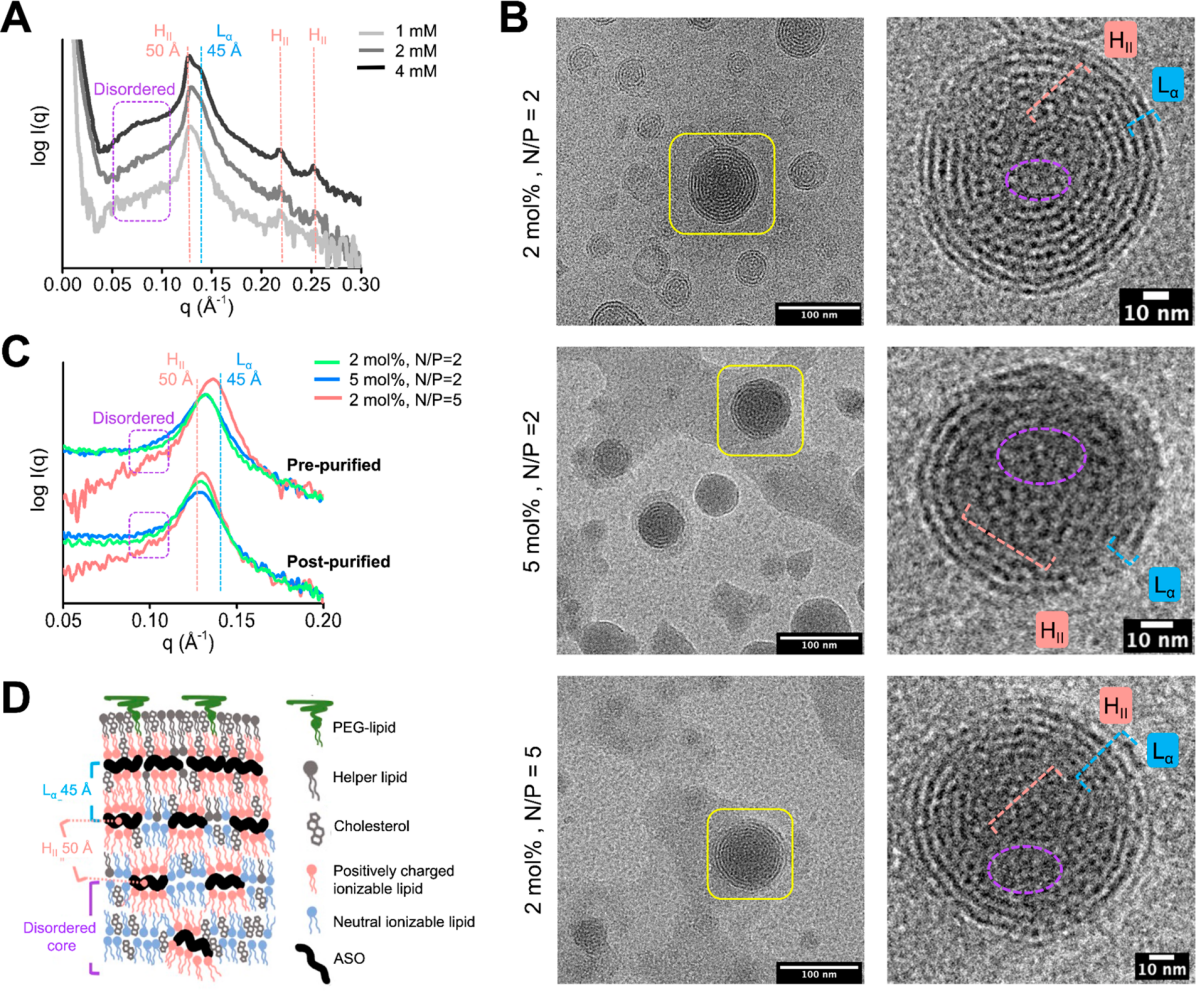
Structure comparison of LNPs with different compositions and buffer conditions. (A) SAXS profile of prepurified LNPs analyzed at different total lipid concentrations. The 1 mM sample was diluted 1:1 in PBS from the 2 mM sample. The position of H_II_ phase peaks at *q*, q*√3, and q*√4 with the spacing *d* = 50 Å and the L_α_ phase peak with *d* = 45 Å are highlighted with red and blue dashed lines, respectively. SAXS signal of the disordered phase with a larger *d* spacing is highlighted with a violet box. (B) Cryo-EM images of prepurified LNPs prepared with 1 mM total lipids. Representative particles in each formulation are magnified to highlight ASO-lipid compartmentalization in the H_II_ and L_α_ phases, which are indicated by red and blue scales, respectively. The violet circle highlights the disordered phase in the core of the particle. (C) SAXS profiles of prepurified (top) and postpurified (bottom) LNPs prepared with different compositions and a total lipid concentration of 1 mM. The positions of SAXS signatures of the H_II_ and L_α_ phases, with the approximate *d* spacing of 50 and 45 Å, are highlighted with red and blue dashed lines, respectively. (D) Proposed distribution of H_II_, L_α_, and disordered phases in the LNP core with highlighted *d* spacing in-between ASO-lipid multilayers (blue spacer) and ASO-lipid compartments (red spacer).

Recently, the solvent injection method using robotic liquid handlers is emerging as an alternative formulation approach. It enables high-throughput generation and screening of LNPs with varied formulation parameters.^14,37^ We have previously demonstrated that the LNPs generated with this approach can be smoothly translated to scalable microfluidic methods in terms of physicochemical properties and *in vitro* performance.^14,17^ Therefore, this high-throughput method was explicitly chosen for all LNP preparations in this work and combined with HT-SAXS^32^ to elucidate structure–activity dependence using a comprehensive data set. In this workflow, we first formulated ASO-LNPs composed of 40 mol % MC3, 2 mol % DMG-PEG-2k, 10 mol % DSPC, and 48 mol % cholesterol at an N/P ratio of 2 to assess SAXS features. As shown in Figure 1A, the LNPs in pre-purification conditions (1:3 volume ratio of ethanol:25 mM citrate buffer, pH 4) exhibited sharp SAXS features, indicating highly ordered LNP morphology. The 4 mM total lipid concentration showed two overlapping but distinguishable peaks at *q* = 0.126 and 0.139 Å^–1^. Using the relationship *d* = 2π/*q*, where *d* is the distance between lipid/ASO/water repeated structures, the peaks show organized structures spaced at *d* = 50 and 45 Å, respectively (Figure 1A). The maxima at *d* = 50 Å is associated with the hexagonal phase (H_II_). Confirming this assignment are the two apparent ancillary peaks at *q* = 0.218 and 0.251 Å^–1^ that would be expected for hexagonal packing at a 50 Å distance. Although the main peak splittings between 50 and 45 Å are less significant at lower (2 and 1 mM) total lipid concentrations (Figure 1A), the asymmetric peak shape indicates the persistence of the H_II_ phase with a multilamellar phase L_α_ with *d* ∼ 45 Å. In addition to the distinct primary peaks, the SAXS curve showed a broad shoulder at *q* = 0.07–0.11 Å^–1^ that can be assigned to the LNP disordered phase.^20,22−24,38,39^ The LNP formulation that does not form an ordered H_II_ phase shows a distinct shoulder at this *q* range (see next section). The disordered phase could be described as a structural precursor of ordered H_II_ phase, as previously shown for multicomponent amphiphilic systems.^40^ Similar to our SAXS profiles, the broad SAXS shoulder at *q* ∼ 0.1 Å^–1^ (*d* ∼ 60 Å) was also observed in coexistence with a sharp peak at *d* ∼ 45–50 Å in siRNA-LNP systems at higher N/P ratios.^24^ In that study, Aburai *etal*. applied a SAXS fitting approach^41^ and determined that a broad shoulder at *d* ∼ 60 Å was related to weakly ordered stack particles in the LNP core. Nevertheless, the contribution of unoriented H_II_ form factor and lamellar form factor may also contribute to the diffuse SAXS signal.^42^

Cryo-EM images of our formulations confirmed our interpretation of peaks resulting from mixed morphologies. The most obvious phase from cryo-EM images is the ordered outer layers of the L_α_ multilamellar morphology with an interlayer spacing of ∼45 Å (Figure 1B). This distance correlates with the SAXS signal of the L_α_ phase. Cryo-EM also showed the presence of ASO-lipid compartments packed in a periodic mesh-like structure and a disordered phase in the particle core (Figures 1B and S1). The repetitive distances between ASO-lipid compartments correspond to the H_II_ phase signature with *d* = 50 Å (Figure 1A). This ASO-lipid compartmentalization in the inverted micelles is generated by the association of ionizable lipids with nucleic acid,^24,26^ where the nucleic acid is stabilized by electrostatic interactions between ionizable lipid headgroups (N) and ASO phosphate groups (P). These hydrophobic compartments aggregate and then become coated with a monolayer of polar lipids such as DSPC and PEG-lipids as these lipids reach their solubility limit in the ethanol/water mixture during the mixing process.^20,27,43^ The repetitive distances between ASO-lipid hexagonal micelles packed in the H_II_ phase and those between multilamellar ASO-lipid layers both contribute to the SAXS signal. LNP size undoubtedly plays a role as for very small LNPs, the repetitive distances between ASO-lipid compartments diminish in small particles due to an increased steric hindrance associated with the high curvature of the particle. Peak broadening reflects a weak distance correlation of the ASO-lipid compartments in the core (Figure 1D). Given the coexistence of the disordered, H_II_, and L_α_ phases, the contributions from each of the three phases will be considered in the subsequent SAXS analyses.

#### Impact of PEG-Lipid Concentration and N/P Ratio on LNP Structure

Using our assignment of SAXS features, we tested how modifying formulation parameters, including the PEG-lipid concentration and N/P ratio, affected these SAXS features (Figure 1B and C). Specifically, we varied the PEG-lipid concentration from 2% to 5% and N/P ratios of ASO-LNPs from 2 to 5. Increasing the PEG-lipid concentration from 2% to 5% resulted in a broader SAXS main peak with lower intensity (Figure 1C, green to blue). These results indicate that higher PEG-lipid ratios result in less ordered ASO-lipid compartments. This effect has also been suggested in previous literature.^18,23^

On the other hand, a higher N/P ratio favored an ordered morphology, as shown by the increased and narrowed SAXS peak signal associated with the H_II_ and L_α_ phases (Figure 1C, red). High N/P ratios facilitate full ionic interaction of ASO phosphate groups with ionizable lipids. These interactions seed ordered ASO-lipid compartmentalization, as shown previously for mRNA-LNPs^20^ and depicted in Figure 1D. The cryo-EM images collected from these same LNP compositions confirm the presence of the three phases (disordered, ordered H_II_ and L_α_) (Figures 1B and S1). Furthermore, both the cryo-EM and dynamic light scattering (DLS) (Table S1) results show overall similar particle sizes among the three formulations, thus excluding particle size as a factor for the interpretation of SAXS signal differences here. For very small particles, the MC3 lipid may disfavor the ordered H_II_ phase because its canonical shape supports negative curvature in the ASO-lipid compartments.

Significant from the results described above, with important implications for our further analysis, is the understanding that the more distinct the H_II_ or L_α_ signatures are from one another, the more the order in the LNP core is distinct from the formation of ASO-lipid multilayers. This is manifested in some of the above results with sharp peaks of varying heights. Later we show formulations where the two peaks separate further from one another in scattering angle. The broadening of these peaks, as observed at a higher PEG-lipid ratio, is linked to a weaker distance correlation between ASO-lipid compartments. Tracking these features provides a means to rapidly measure the internal structure of LNPs.

#### Conformational Transition during Buffer Exchange

We next examined the potential of LNP morphology changes after purification. During the purification process, the prepurified LNPs are buffer exchanged into a physiologically relevant phosphate-buffered saline (PBS) buffer with pH 7.4. This process is to remove ethanol, excess lipids, and the nonencapsulated nucleic acid cargo. The most important change is the increased buffer pH from 4 to 7.4. The pH change neutralizes the cationic charges on ionizable lipids,^20^ which may cause LNP structural transition.^20−22^ To examine this effect, we compared the structure change of three LNPs with different N/P ratios and PEG-lipid concentrations before and after purification (Figure 1C). The particle size of the three investigated LNPs remained constant throughout the purification process (Table S1). However, we observed small changes in features going from pre- to postpurified LNPs, with signatures of an increasing hexagonal phase shifting the primary peak position (Figure 1C). After purification by buffer exchange, an overall increase in internal ASO-lipid compartmentalization could also be observed by cryo-EM (Figures 1B and S2), contributing to the shifted H_II_ peak signature. This was more obvious for the LNP with N/P = 5, which retained a lower amount of disordered and a higher amount of L_α_ phases, compared with N/P = 2 conditions. Under high N/P ratios and an acidic buffer pH, excess positive lipid charges may repulse the electrostatic interaction between the charged lipids and the nucleic acid cargo in the particle core. Balancing these excess lipid charges by increasing the buffer pH has been shown to change the internal structure of lipid-nucleic acid packing.^44^ In line with recently published results,^20^ water influx-mediated particle swelling, or particle size increase, during pH increase was insignificant for LNPs composed of saturated phospholipids, e.g., DSPC used in the LNPs investigated in our study.

The differences among the three postpurified LNP formulations were smaller than in prepurification conditions, but the trends were consistent. Purification by buffer exchange adds another processing step in preparing LNPs, introducing additional time-dependent factors. LNPs in their prepurification buffer represent the earliest consistently reproduced state right after the high-throughput formulation preparation. In the following studies, we focused our analysis and comparisons on prepurified LNPs, as this state is critical in the early stage screening and development of LNPs. Also, the structural trends of purified LNPs will depend on those observed in prepurified states.

### PEG-Lipids Alter ASO-LNP Structure that Controls *in Vitro* Efficacy

We next utilized the results described above to establish a SAXS structural elucidation workflow that allows for quick and efficient structure–function correlation analyses. To streamline this effort, we focused our structural studies on a set of ASO-LNP formulations containing PEG-lipid compositions that were previously assessed for both particle size and cellular *TMEM106b* mRNA knockdown efficacy.^17^ While this study found that PEG-lipid molar ratio controls particle size and PEG-lipid carbon tail length regulates ASO-LNP gene silencing activity in murine cortical neurons, the impact of structure was not assessed. Thus, we next built upon this data by using HT-SAXS^32^ to examine the internal structures of these 54 ASO-LNP formulations. We also selected representative samples to further characterize using cryo-EM. Ultimately, we determined the key LNP features that predict *in vitro* efficacy.

#### PEG-Lipids Alter ASO-LNP Internal Structure

**Figure 2:**
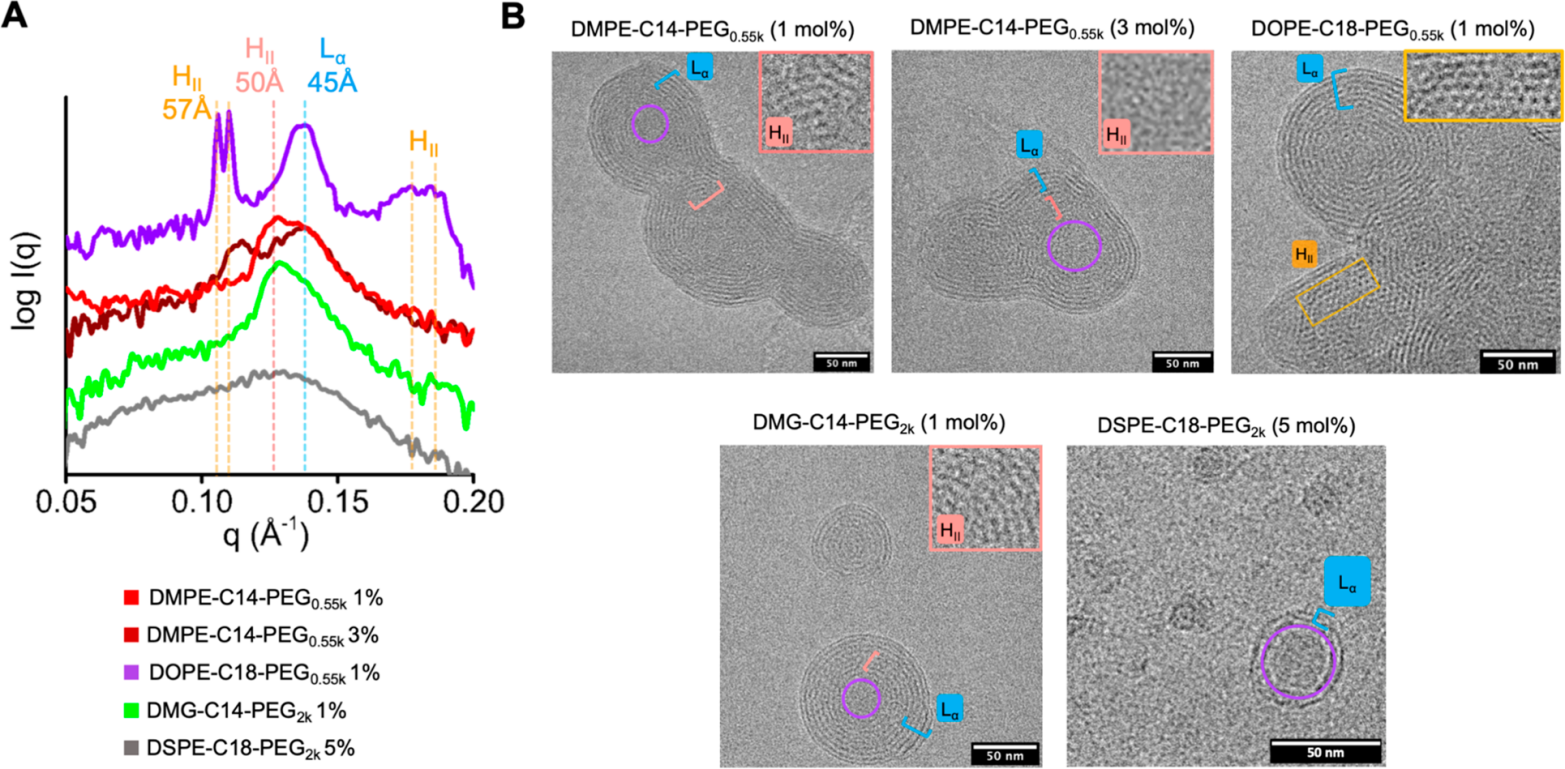
PEG-lipid characteristics affect LNP structure. (A) SAXS analysis of 5 LNPs with different PEG-lipids composition. The red and blue dashed lines indicate the H_II_ and L_α_ signal positions as assigned in Figure 1A. Primary and secondary H_II_ diffraction peaks at *q* ∼ 0.11 and ∼ 0.18 Å^–1^, respectively, potentially associated with the crystalline phase, are highlighted with orange dashed lines. (B) Cryo-EM images of LNP formulations investigated in panel A. The cryo-EM images highlight the compartmentalization of the ASO-lipid in the H_II_ (red scale), crystalline H_II_ (yellow box), L_α_ (blue scale), and disordered phases (violet circle). ASO-lipid compartmentalization in the H_II_ phases of some LNPs is highlighted in the zoom-in window.

The ASO-LNP formulations were prepared with a total lipid concentration of 1 mM and an N/P ratio of 2 using MC3, DSPC, cholesterol, and PEG-lipid analogs under a molar ratio of 40:10:(50 – *X*):*X*, where *X* = 1, 3, or 5. A diverse set of SAXS curves was generated to establish the relevance of 54 LNP formulations (Figure S3). Five representative formulations with distinct SAXS features were chosen for cryo-EM imaging. A comparison of the SAXS curves and cryo-EM images of these distinct samples showed the dramatic differences in the LNP morphology (Figure 2) depending on the PEG-lipid type and its molar ratio. LNPs with 1 mol % of DMPE-C14 PEG_0.55k_ showed a broad primary SAXS peak (Figure 2A, red) with two maxima associated with the H_II_ and L_α_ phases, respectively. Cryo-EM images of this sample showed ASO-lipid compartmentalization in the core and multilamellar layers surrounding the core (Figures 2B and S4). A significant change occurred by increasing the DMPE-C14 PEG_0.55k_ molar ratio to 3%. The H_II_ peak was shifted toward a larger *d* spacing of ∼55 Å, while the L_α_ signature remained unchanged at *d* = 45 Å (Figure 2A, dark red). Cryo-EM showed the presence of larger nonspherical LNPs with distinct compartmentalization of ASO-lipid complexes (Figures 2B and S4). The shift of the H_II_ signature reflects an increasing order of ASO-lipid compartments. LNPs formulated with 1 mol % of DMG-C14 PEG_2k_ showed a weaker L_α_ signature, indicating less abundant multilayer morphology (Figure 2A, green). This correlated with a relatively smaller LNP size with 1 mol % DMG-C14 PEG_2k_ than that with 1 mol % DMPE-C14 PEG_0.55k_ (diameter of 117 nm vs 190 nm, Table S2). Cryo-EM results also showed smaller particles with fewer fused multilayers in the former LNP formulation (Figures 2B and S4). Compared to DMPE-C14-PEG_0.55k_, DMG-C14 PEG_2k_ has a longer PEG unit and does not have the phosphoric group in the linker chemistry.

Very large particle sizes (diameter >200 nm, Table S2) were observed for LNPs formulated with 1% DSPE-C18 PEG_0.55k_ and DOPE-C18 PEG_0.55k_, short PEG units. Also, the H_II_ signature was shifted even more than for the DMPE-C14 PEG_0.55k_. Both formulations showed two H_II_ diffraction-like peaks around *q* ∼ 0.11 Å^–1^ (*d* ∼ 57 Å) with a further ancillary peak associated with hexagonal packing at *q* ∼ 0.18 Å^–1^ (Figure 2A, violet; Figure S3). The L_α_ signal remained at *d* = 45 Å. The cryo-EM results showed highly ordered compartments of ASO-lipids and an abundant multilamellar phase (Figures 2B and S4). The signals observed from the low molar ratio of PEG-lipids with a short PEG unit and a charged phosphoric group are consistent with a colloidal instability. Fusion of the unstable LNPs forms very large LNPs that allow the packing of ASO-lipid into the crystalline-like H_II_ phase. Notably, low cholesterol solubility within the surface DSPC/PEG-lipid monolayer may lead to LNP instability that supports crystal-like compartmentalization of ASO-lipid in large particles. Indeed, the diffraction peak at *q* ∼ 0.18 Å^–1^ was previously assigned to the crystal-like phase in the cholesterol/MC3/polyA mixture.^23^ The limited solubility of cholesterol^45^ in the surface DSPC/PEG-lipid bilayer was shown to support the formation of cholesterol crystalline domains and, consequently, a fusion of LNPs into larger-sized particles.^23^ As expected, higher ratios of DOPE-C18 PEG_0.55k_ and DSPE-C18 PEG_0.55k_ reduced signals from the crystalline phase. The H_II_ diffraction peak at *d* ∼ 57 Å changed into a broad peak with the disappearance of secondary peaks (Figure S3). This indicates that higher molar ratios of certain PEG-lipids with a short PEG length are essential to allow higher solubility of cholesterol in the surface PEG-lipid layer and lead to more stable LNPs. In addition, the PEG shielding may be less efficient with short PEG units; therefore high molar contents of these PEG-lipids may be required to maintain the colloidal stability of LNPs.

These results show that the SAXS signal can be used to identify unstable LNP formulation conditions that could be excluded from subsequent functional analysis. Formulations at high concentrations of PEG-lipids with long PEGs yielded LNPs with the greatest contrast to the near crystalline formulations described above. For example, the LNP composed of 5 mol % DSPE-C18 PEG_2K_ showed a wide SAXS signal, lacking distinctive phase-associated peaks (Figure 2A, gray). This indicates small particles with a disordered internal morphology, as was further visualized by cryo-EM (Figure 2B).

#### Quantifying ASO-Lipid Compartmentalization Using SAXS Peaks

**Figure 3:**
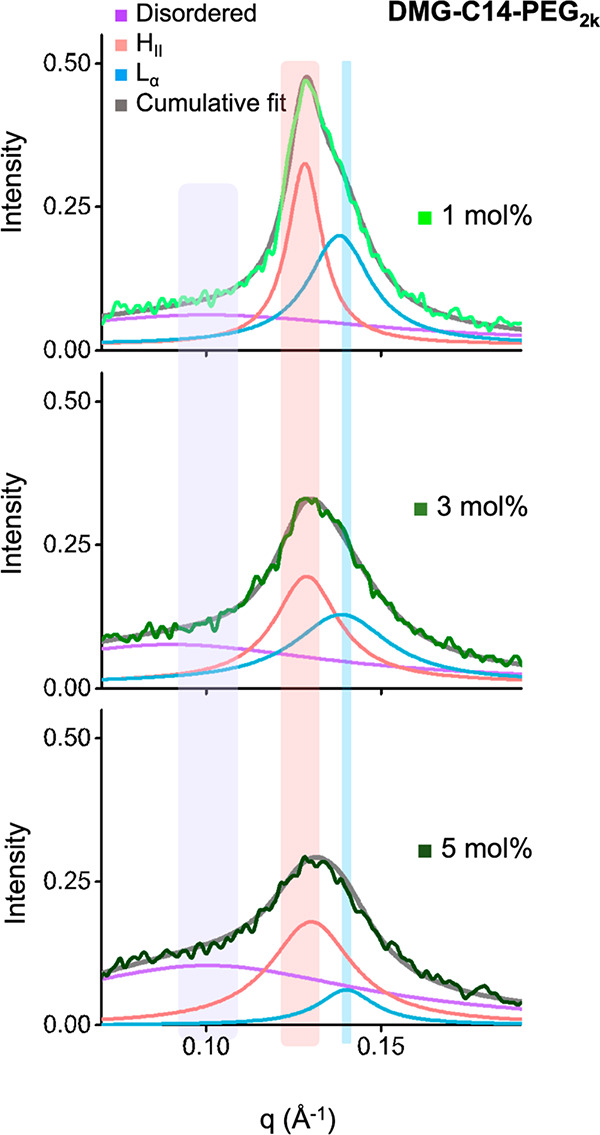
Deconvolution of SAXS peaks correlates to ASO-lipid compartmentalization in the LNP core. Deconvolution of primary SAXS peaks of a representative LNP formulation using three Lorentz functions associated with disordered, H_II_, and L_α_ phase signals. The center of Lorentz functions and its lower and upper bounds used in the fitting of the three phases are highlighted with violet, red, and blue stripes, respectively. See Materials and Methods section for detailed description of the fitting approach.

To quantitatively characterize the relative contributions of H_II_, L_α,_ and disordered phases in LNP formulations and determine the level of ASO-lipid compartmentalization, we fit the primary SAXS peak of 52 formulations (excluding the two unstable LNPs in the screening library as discussed above) with three Lorentz functions centered at *d* ∼ 45, ∼ 50, and ∼60 Å, respectively (see Methods, Figures 3 and S5). Using this approach, we observed that model fitting of the higher molar ratio PEG-lipid conditions generally required broader Lorentz functions than those used to fit the lower ratios. For example, the fit to DMG-C14 PEG_2k_ formulation (Figure 3) as a function of molar ratio showed diminishing H_II_ and L_α_ phases and an elevated disordered signature. This indicates that higher PEG ratios create less ordered LNP structures.

#### Particle Size Correlates with Multilamellar Structure

**Figure 4:**
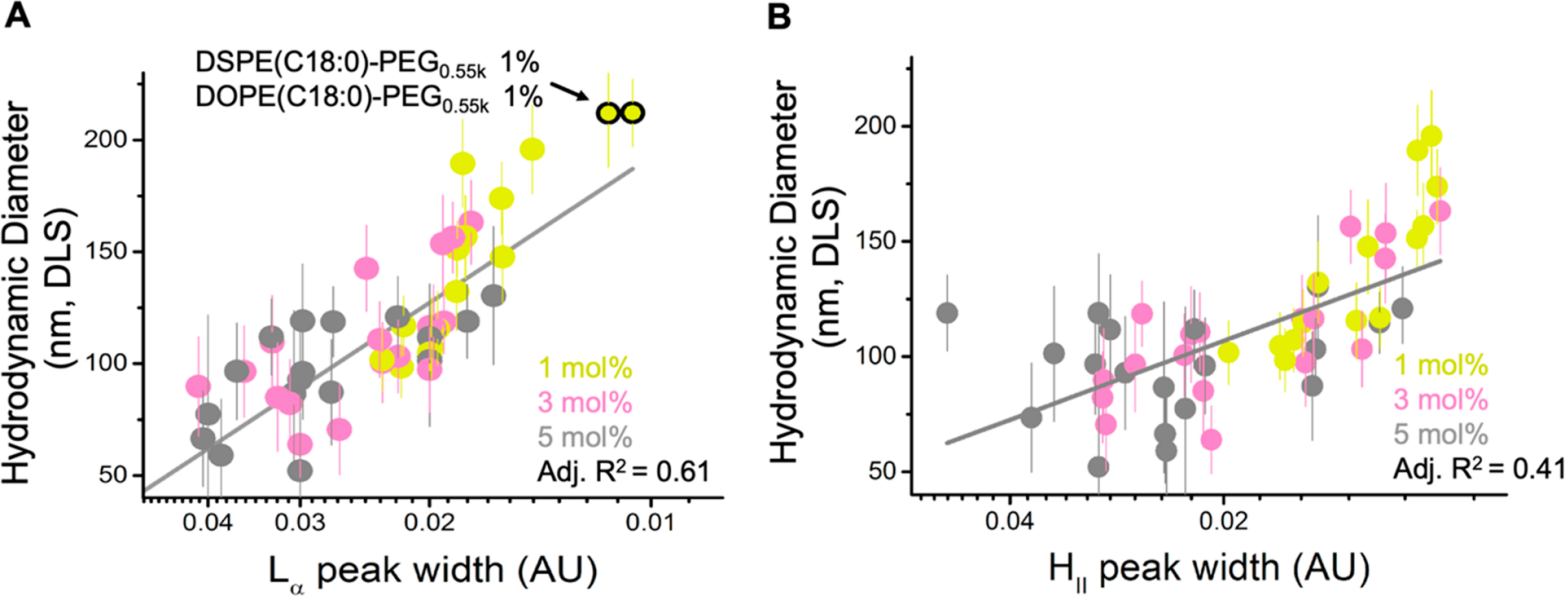
Multilamellar structure of LNP surface layers correlates with the particle size. Correlation between the Lorentz function width of the L_α_ (A) or H_II_ (B) signal (shown in log scale to better indicate data distribution) with particle size determined by DLS across the library of 54 LNP formulations. See the Materials and Methods section for derivation of Lorentz function width. The molar percentage of PEG-lipids is colored accordingly. Unstable LNP formulations (with 1% DSPE-C18 PEG_0.55k_ or 1% DOPE-C18 PEG_0.55k_) showing no H_II_ signal are highlighted by black circles in panel (A) and are excluded from the analysis in panel (B).

To test whether and how internal morphology is linked to overall LNP size, we next examined the correlations between the deconvolved SAXS peaks (H_II_, L_α_, and disordered) and the hydrodynamic particle diameter determined by DLS (data reprised from ref (17)). Broad L_α_ peak widths correlated with smaller LNP diameters, with a linear correlation *R*^2^ of 0.61 (Figure 4A), connecting the prevalence of disordered internal morphologies to smaller LNPs. Broad L_α_ peaks were also associated with higher PEG ratios. Narrow L_α_ width associated with repeated multilamellar morphology was observed more prominently for larger LNPs with 1 mol % PEG-lipid. In contrast, we observed a weaker correlation between H_II_ peak width and LNP size, with a linear correlation *R*^2^ of 0.41 (Figure 4B). Narrow H_II_ peak width was also more associated with LNPs with 1 mol % PEG-lipids than LNPs with higher PEG molar ratios. Our results agree with previous studies showing that PEG-lipids in LNP formulations regulate particle sizes during self-assembly.^14,39,46−48^ Furthermore, the structural analysis by SAXS indicates that the multilamellar structure near the particle surface, correlating to the L_α_ phase, occupies most of the particle volume of large LNPs and primarily drives the particle size differences, while particle size is less correlated with the structural features toward the particle core area (H_II_ and disordered phases).

#### *In Vitro* Efficacy Predictably Correlates with ASO-LNP Core Structure

**Figure 5:**
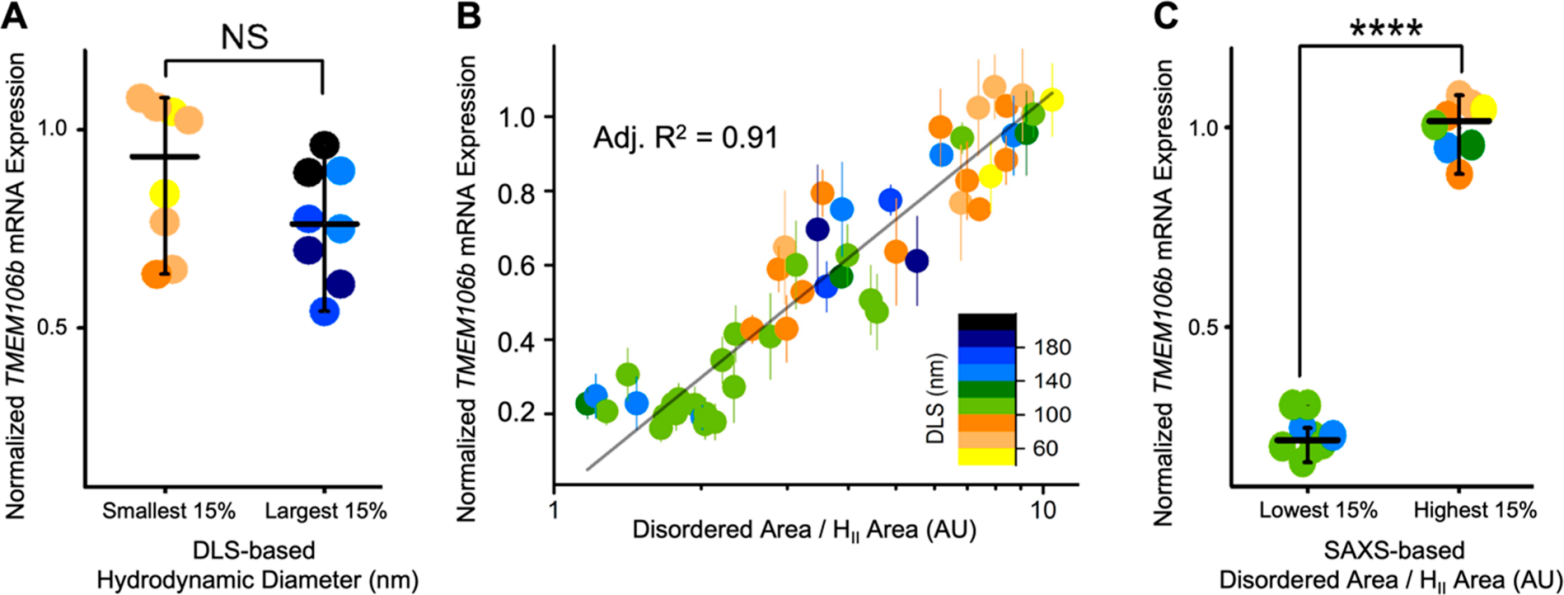
LNP structure determined by SAXS features relates to *in vitro* efficacy. (A) DLS-based LNP sizes do not show a significant correlation with relative *TMEM106b* mRNA expression that can be predictively used for hit identification. (B) Correlation between disordered/H_II_ Lorentz function area ratios and relative *TMEM106b* mRNA expression across the library of 54 LNP formulations. See the Materials and Methods section for derivation of Lorentz function area. The data are colored based on the LNP size measured by DLS (inset legend). (C) A comparison between the bottom and top 15% of the ratio values and relative *TMEM106b* mRNA expression suggests an ordered LNP core character results in efficacious formulations.

The 54 ASO-LNP formulations characterized in this study were previously assayed by Sarode *etal*. for *TMEM106b* gene silencing activity in murine cortical neurons using qRT-qPCR, which reads out ASO delivery by measuring changes in cellular mRNA expression.^17^ Lower mRNA expression indicates stronger ASO-mediated knockdown efficacy. Our previous report showed that while there was a correlation between *in vitro* ASO efficacy and PEG-lipid tail length, no correlation was observed with PEG molecular weight or molar ratios.^17^ As PEG-lipids have been widely reported to affect LNP size, we first examined whether *in vitro* ASO activity correlated with LNP diameter. When comparing the largest and smallest ASO-LNPs in our library (Figure 5A), LNP diameter was not predictive of ASO delivery, showing very little correlation to *in vitro* efficacy. We next investigated whether the predominance of phases, internal to LNPs, correlated with ASO-LNP activity.

Following extensive regression analysis, the deconvolved peak area ratio of disordered to H_II_ type order in the LNP core was found to be the most decisive structural indicator of ASO-LNP efficacy. In particular, SAXS peak area ratios reflect the relative contribution of different structural organizations and also mitigate potential concentration differences among samples. This ratio correlated to ASO activity in cortical neurons with an *R*^2^ value of 0.91 (Figure 5B) and reflects the distribution of disordered to ordered (H_II_) ASO-lipid compartmentalization in the LNP core. Among the 54 ASO-LNP formulations screened, those with the lowest disorder-to-order ratio values showed about a 4-fold increase in ASO efficacy compared to those with the highest ratio values (Figure 5C). We note that neither the L_α_ nor the H_II_ signature peak width, which respectively showed strong and weak negative correlations with the LNP size (Figure 4), correlated with *in vitro* ASO activity (Figure S6A,C). The peak area ratio between the disordered core and L_α_ lamellar phase also did not correlate with *TMEM106b* mRNA expression (Figure S6B). These comparisons revealed that ASO-LNP efficacy is governed by the core compartmentalization between disordered to ordered (H_II_) morphology. An ordered LNP structure is recognized as one of the critical components promoting endosomal escape,^49−51^ which is considered as one of the critical factors determining the cellular LNP efficacy.^23,52^ More recent literature also reported similar findings related to impacts of the order within the LNP core on mRNA delivery efficiency.^37,44^ Our finding that an ordered particle core favors enhanced mRNA knockdown provides insights into functional delivery and can be utilized to further optimize LNP formulations.

#### Improved Efficacy by Tuning Chemical Composition of PEG-Lipids

**Figure 6:**
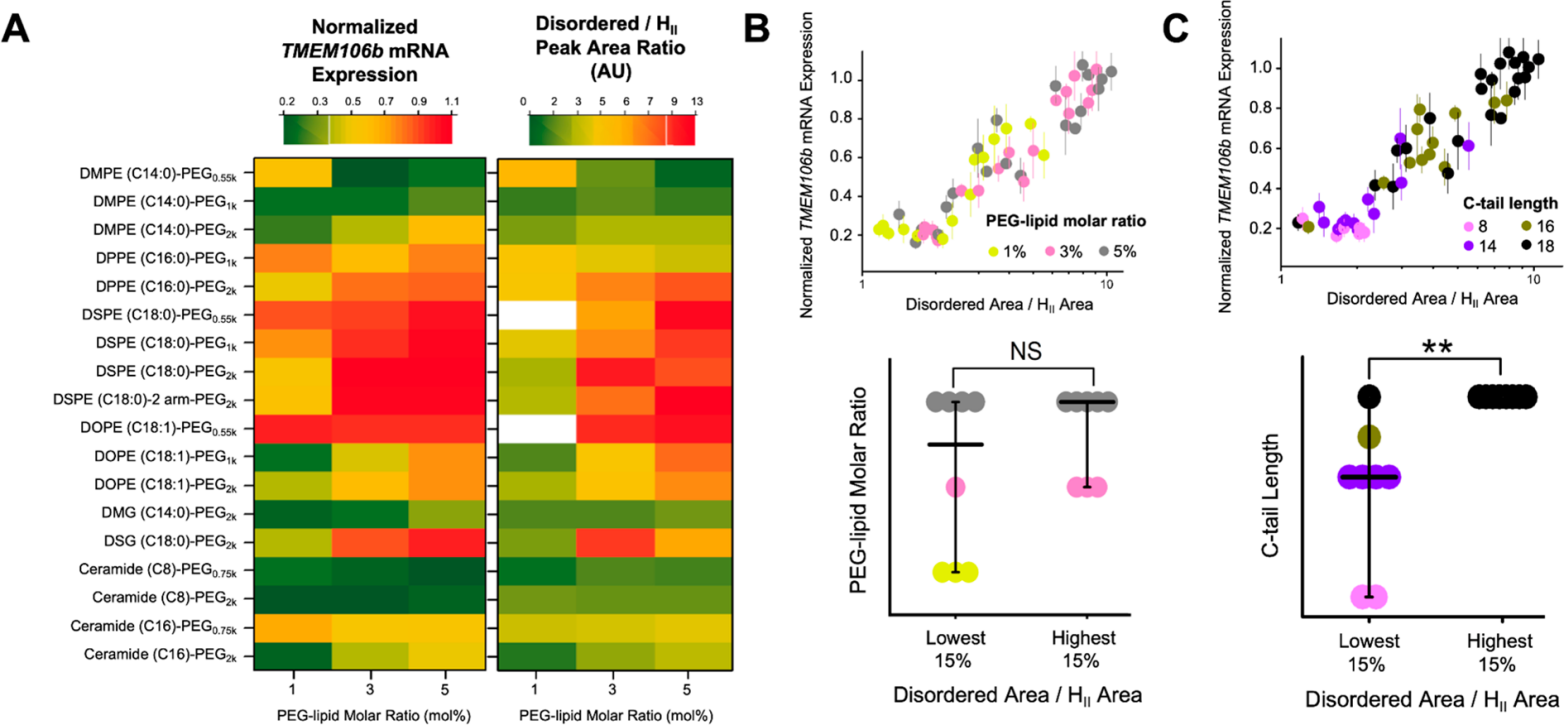
PEG-lipid properties impact internal structure and cellular knockdown efficacy of ASO-LNPs. (A) The 54 formulations are presented using color-coded heat maps to rank-order them based on the relative mRNA expression (left panel) and disordered/H_II_ peak area ratios (right panel). Two unstable formulations without the primary H_II_ peaks were excluded from the SAXS model fitting and are indicated in white in the SAXS ratio heat map. (B, C) Correlations among disordered/H_II_ peak area ratio, PEG-lipid carbon tail length, PEG-lipid molar ratio, and relative mRNA expression across the screening library. **p < 0.05, ***p < 0.001, ****p < 0.0001, NS: nonsignificant, as analyzed by two-tailed *t* tests. All cellular efficacy and DLS data were obtained from our previous study.^17^

We next used a heat map to visualize the properties of PEG-lipids that improve core organization, thus yielding improved ASO-LNP transfection. Side-by-side comparisons of mRNA expression levels and the predictive ratio extracted from SAXS depict the structural correlation previously described while simultaneously revealing the impact of PEG-lipid composition (Figure 6A). The two PEG-lipids that resulted in very large LNPs with crystalline internal structures and poor *in vitro* mRNA knockdown are indicated in white in the SAXS ratio heat map.

The most organized LNP cores were formed with low molar ratios of PEG-lipids with short diacyl tails (e.g., DMPE-C14, Ceramide C8). ASO-LNPs formulated using DMPE-C14 PEG with a PEG size of 1 kDa adopted an ordered H_II_ phase and showed desirable efficacy for PEG-lipid ratios ranging from 1 to 5 mol % (Figure 6A). The same DMPE conjugated with a shorter PEG (0.55 kDa) required higher PEG-lipid ratios (3 and 5 mol %) to form the H_II_ phase and consequently increase ASO-mediated mRNA degradation. However, in general, the molar ratios of PEG-lipids did not correlate with disordered/H_II_ peak area ratios (Figure 6B). Higher percentages of DMPE conjugated with a longer PEG (2 kDa) formed smaller LNPs with a disordered core, thus significantly reducing ASO-LNP activity.

While the influence of cationic lipids on nucleotide organization within the LNP core is appreciated, here we also observed large changes in LNP core organization due to the use of different PEG-lipids. PEG-lipids are often introduced to adjust interparticle interactions between LNPs and surface interactions of LNPs with serum proteins and cells. In our previous study, we observed that the shorter carbon tail length of PEG-lipids correlated with stronger gene silencing activity of ASO-LNPs. This was presumed partially due to faster shedding of shorter carbon tails of PEG-lipids from LNP surfaces, leading to enhanced cellular interaction.^17^ Here, from an LNP structure perspective, our in-depth SAXS analysis reveals a strong correlation between the disordered/H_II_ peak area ratio and the carbon tail length of PEG-lipids (Figure 6C). This indicates that PEG-lipids also substantially impact LNP internal structure. Short carbon tails of PEG-lipids produce more ordered LNP cores that potentially promote endosomal escape of the cargo, therefore contributing to enhanced cellular efficacy.

LNP internal morphology was also affected by the PEG-linker charge and chemistry. For example, LNPs with the ceramide C16-PEG_2k_ showed a favorable decrease in mRNA expression and a substantially ordered core, while the DSPE-C18 PEG_2k_ showed reduced efficacy and a mostly disordered core (Figure 6A). In addition, ASO-LNPs prepared with the neutral and short alkyl chain ceramide (Ceramide-C8 PEG_0.75 or 2k_) or the neutral diglyceride PEG-lipid (DMG-C14 PEG_2k_) showed weak dependence of SAXS ratios on their molar content ratios. These LNPs adopted highly ordered core structures, constant particle sizes (Table S2), and favorable gene silencing activity across 1–5 mol % of PEG-lipids.

In summary, our results show that SAXS can efficiently monitor LNP internal morphology. Our results also elucidate that the compartmentalization of ASO-lipid in an ordered LNP core is essential for strong mRNA knockdown *in vitro*. The disordered/H_II_ peak area ratio is identified to quantitatively predict efficacy readouts, thus allowing LNP formulation optimization based on structure–activity relationships.

## Conclusions

While LNP morphology is recognized as a critical parameter governing LNP bioactivity, structural analysis is typically not assessed due to limited accessibility and resource requirements. Therefore, an understanding of LNP SAR has been lacking in data sets published to date. To address this issue, we integrated HT-SAXS into our formulation screening platform to create an HTS workflow that encompasses ASO-LNP formulation, physicochemical property evaluation, structural analysis, and *in vitro* gene silencing assessment. This seamless workflow enabled the acquisition of a substantial data set that allowed for thorough examination of LNP SARs.

**Figure 7:**
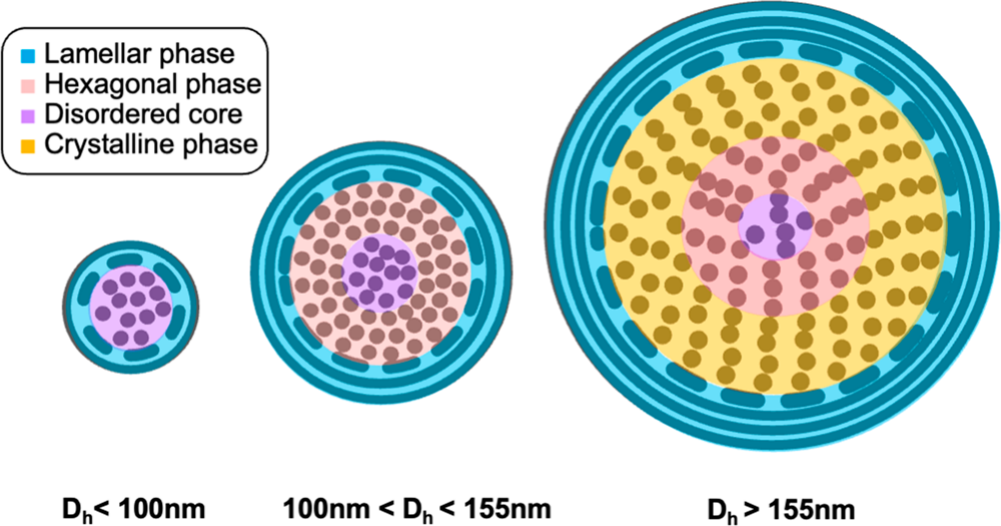
Cellular efficacy of ASO-LNPs is governed by the extent of order within their core. The various structural features are shown in four different colors. The dots represent the ASO-lipid compartmentalization, and the circles represent the ASO-lipid bilayers. The medium-size LNPs with a hydrodynamic diameter (*D*_h_) of 100–155 nm and low nonorder-to-order ratios (disordered/H_II_) show the best *in vitro* efficacy. Small LNPs (*D*_h_ < 100 nm) feature abundant disordered phases but lack the ordered structure in the particle core, whereas very large LNPs (*D*_h_ > 155 nm) show unstable crystalline structure with reduced hexagonal phase in the particle core. Both scenarios lead to high disordered/H_II_ ratios, correlating with diminished *in vitro* efficacy.

In this study, we prepared 54 ASO-LNPs based on the screening libraries of PEG-lipids for the comprehensive structural analysis. The SAXS peak assignments were verified by cryo-EM with representative LNP samples. To further achieve a quantitative structure comparison, the Lorentz functions were adapted to deconvolve the primary SAXS peaks into three compartments, representing disordered, H_II_, and L_α_ particle core features (Figure 7). Additionally, SAXS was able to easily identify unstable LNPs forming an ordered crystalline H_II_ phase that significantly diminished efficacy. Our established model revealed a strong correlation between *in vitro* efficacy and the transition from a nonordered to an ordered structure presented in the LNP cores. This model successfully predicted *in vitro TMEM106b* mRNA knockdown performance of the 54 LNP samples in murine cortical neurons. Our result is in agreement with previous findings showing that an ordered oligonucleotide-lipid phase promotes oligonucleotide endosomal escape and increased efficacy. Our developed analysis can be applied to characterize LNPs at various stages during preparation, storage, and delivery. Taken together, our workflow can be used to develop LNP SAR and allows for the identification of critical formulation parameters in the vast formulation space, which can subsequently be used to optimize LNP performance.

Our approach complements other methods used to predict LNP performance, which has historically focused on conventional physicochemical property assessments, including particle size distribution, encapsulation efficiency, and stability in serum. While we applied our efforts to build an SAR for ASO-LNPs in murine cortical neurons, this correlation could be further investigated for additional therapeutic targets in other cell types of interest. This workflow could also be applied to other nucleic acid-LNPs, including siRNA and mRNA, to explore LNP SARs with various modalities.

## Materials and Methods

### Materials

Lipids, including cholesterol, DSPC, and PEG-lipids, except the DSPE-2 arm-PEG_2k_ were purchased from Avanti Polar Lipids (AL, USA). The two-arm PEG lipid was from NOF (NY, USA). The ionizable lipid DLin-MC3-DMA was procured from MedChemExpress (NJ, USA). The *TMEM106b*-targeting 17-mer ASO (MW 5635 g/mol) was custom synthesized by BioSpring GmbH (Frankfurt, Germany) in the Na salt form with a phosphonothioate backbone, following standard phosphoramidite protocols as described previously.^53^ The ASO sequence (base sequence, sugar sequence) is the following: ATGTACTAATTTTETTT, LLLLDDDDDDDDDLLLL. Mouse embryonic cortical neurons were prepared from animal tissues and cultured as described previously.^17^ All other reagents were DNase/RNase-free.

### High-Throughput Formulation and Characterization of LNPs

Individual ASO-LNP samples and the screening sample plates were prepared by a robotic liquid handler-assisted, high-throughput solvent-injection method that we developed previously.^14^ Briefly, a liquid handler (TECAN EVO, NC, USA) was used to dispense the ASO dissolved in a citrate buffer (25 mM, pH 4) into a 96-well sample plate, as well as to mix individual lipid stocks at certain molar ratios to generate different lipid mixtures. For the PEG-lipid screening, LNPs were designed with the lipid composition with molar percent of MC3:DSPC:cholesterol:PEG-lipids at 40:10:(100 – *X*):*X*, where *X* = 1, 3, or 5, a total lipid concentration of 1 mM, and an N/P ratio of 2. The prepared lipid mixtures were rapidly mixed with the ASO aqueous phase at a volume ratio of 1:3 (50 μL:150 μL) in the sample plate using the robot, allowing for self-assembly of ASO-loaded LNPs, which were then characterized for particle structure by SAXS in the preparation buffer and particle size distribution by DLS (Wyatt DynaPro Platereader III, CA, USA) after 50× dilution in PBS. In separate experiments, certain LNPs were processed through ultracentrifugation using an Amicon filter with the MWCO of 10 kDa (Millipore, MA, USA) for purification and buffer exchange to PBS. Aliquots of LNPs were diluted in PBS and then culture media to a total ASO dose of 50 nM for *in vitro* knockdown efficacy screenings in neural cell cultures, as detailed previously.^17^

### SAXS Data Collection and Analysis

SAXS data were collected in the high-throughput mode (HT-SAXS) using the Advanced Light Source SIBYLS beamline 12.3.1 at the Lawrence Berkeley National Laboratory (CA, USA). X-ray wavelength was set at λ = 1.216 Å, and the sample-to-detector distance was 2070 mm, resulting in a scattering vector, *q*, ranging from 0.01 to 0.45 Å^–1^. The scattering vector is defined as *q* = 4π sin θ/λ, where 2θ is the scattering angle. Experiments were performed at 20 °C as described elsewhere.^32^ Briefly, the sample was exposed for 10 s with the detector framing at 0.3 s to maximize the signal while merging the SAXS signal using the SAXS FrameSlice application (https://bl1231.als.lbl.gov/ran). No radiation damage was observed during the 10 s exposure, and all collected frames were merged. The merged SAXS profile was plotted using OriginPro 2015 (OriginLab Corporation, MA, USA).

Data analysis was performed using OriginPro 2015. The initial positions of primary and secondary H_II_ peaks were defined as *q*1, *q*2 = √3*q1, and *q*3 = √4*q1. The position of the L_α_ peak at *q* ∼ 0.139 Å^–1^ (*d* ∼ 45 Å) was approximate from the SAXS signal measured for ASO-LNP prepared at a total lipid concentration of 4 mM. From the position of the first-order peak *q*1, the corresponding repeat distance of a multilamellar structure was calculated using the Bragg equation: *d* = 2π/*q*1. Deconvolution of the primary SAXS peaks by Multipeak Lorentz fits was performed by OriginPro 2015 in batch mode using the built-in function *y* = *y*0 + (2*area/π)*(width/(4*(*x* – *xc*)^2^ + width^2^)). *y*0 is an offset and was set to 0. *xc* is a center of function and corresponds to the center of the SAXS peak assigned to the disordered, H_II_, or L_α_ phase, respectively. The upper and lower bounds of *xc* were set to a *q* of 0.09–0.11 Å^–1^ for disordered, 0.12–0.128 Å^–1^ for H_II_, or 0.137–0.139 Å^–1^ for the L_α_ signal. The width and area of the functions were not restrained. All SAXS curves are available at simplescattering.com under entries XSSOSU8C, XSDM4ADX, and XSK1IXUI.

### Cryo-EM Imaging

All cryo-EM grids were discharged for 9 s on CEMRC GloQube at 20 mA and prepared on the CEMRC Vitrobot Mark IV, which was set to 4 °C/95% humidity. Freshly prepared LNPs were imaged at the original sample concentration. Excess liquid was blotted using a blot force of −25, wait time of 45 s, and drain time of 0.5 s for all grids. All images were collected on the Talos Arctica 200 keV TEM equipped with a Falcon III camera. Images were collected from holes distributed across overview maps. Selected holes were imaged at higher magnification with a 0.96 Å pixel size and a defocus range of −1.5 to −2.5 μm, or a 0.78 Å pixel size and a defocus of −1.5 μm. Total electron dose was 60 e^–^/Å^2^.

### Statistics

Data analysis was performed using OriginPro 2015. The statistic of the linear fits was analyzed using adjusted *R*^2^. Significance tests were analyzed using paired *t*-tests. All statistical analyses were two-sided and a *P* value < 0.05 was considered as statistically significant.

## Supporting Information Available

Physiochemical properties of ASO-LNPs; SAXS data of all ASO-LNPs samples; additional cryo-EM images; deconvolution of SAXS peaks of representative LNP formulations; correlations between additional SAXS peak measures (PDF)
