# Supplementary material for: Correlating the Structure and Gene Silencing Activity of Oligonucleotide-Loaded Lipid Nanoparticles Using Small-Angle X-ray Scattering
Source: ACS Nano. 2023 Jun 6;17(12):11454–65. doi: 10.1021/acsnano.3c01186 (PMC10311593; doi:10.1021/acsnano.3c01186)
Supplement: Supplementary file 1 — nn3c01186_si_001.pdf [file nn3c01186_si_001.pdf]

Supporting Information

Correlating the Structure and Gene Silencing Activity of Oligonucleotide-Loaded Lipid Nanoparticles Using Small-Angle X-Ray Scattering

Michal Hammel*†[a], Yuchen Fan†[b], Apoorva Sarode†[b], Amy E. Byrnes[c], Nanzhi Zang[b], Ponien Kou[b], Karthik Nagapudi[b], Dennis Leung[b], Casper C. Hoogenraad[c], Tao Chen[b], Chun-Wan Yen*[b], Greg L. Hura*[a,d]

[a] Molecular Biophysics and Integrated Bioimaging Division, Lawrence Berkeley National Lab, Berkeley, CA, 94072 USA

[b] Small Molecule Pharmaceutical Sciences, Genentech Inc., South San Francisco, CA, 94080 USA

[c] Department of Neuroscience, Genentech, Inc., South San Francisco, CA, 94080 USA

[d] Chemistry and Biochemistry Department, University of California Santa Cruz, Santa Cruz, CA, 95064 USA

† These authors contributed equally to this work
[*] Corresponding authors, Email: mhammel@lbl.gov, yenc3@gene.com, glhura@lbl.gov

Table S1. Composition, formulation process and particle size characterization of LNPs shown in **Figure 1**.

LNP lipid composition	N/P ratio	Total lipid concentration (mM)	Formulation process	Mean diameter (nm)	Mean %PD
MC3:DSPC:cholesterol:DMG-PEG$_{2k}$ = 40:10:38:2	2	4	Unpurified	169.8	16.3
		2	Unpurified	132.9	15.5
		1	Unpurified	97.3	15.1
			Purified	97.2	14.1
MC3:DSPC:cholesterol:DMG-PEG$_{2k}$ = 40:10:38:5	2	1	Unpurified	91.6	18.7
			Purified	90.3	22.8
MC3:DSPC:cholesterol:DMG-PEG$_{2k}$ = 40:10:38:2	5	1	Unpurified	91.9	19.2
			Purified	91.9	17.8

Table S2. DLS-based particle size distribution and Oligreen-based ASO encapsulation efficiency of HTS LNPs prepared with 1 mM total lipids, N/P = 2, different PEG-lipid species, and different PEG-lipid molar ratios. (DLS data reprised from our previous publication [17])

PEG-lipid molar ratio→	1%	3%	5%	1%	3%	5%	1%	3%	5%
PEG-lipid type	Hydrodynamic diameter (nm)			% Polydispersity			% Encapsulation efficiency		
DMPE (C14:0)-PEG$_{0.55k}$	189.5	142.6	119.1	10.3	13.5	21.4	83.1	78.5	75.9
DMPE (C14:0)-PEG$_{1k}$	151.3	110.7	101.3	8.0	15.3	28.9	84.5	74.0	70.2
DMPE (C14:0)-PEG$_{2k}$	101.7	89.8	73.5	13.6	24.7	32.1	80.7	68.7	54.2
DPPE (C16:0)-PEG$_{1k}$	173.8	116.5	96.0	9.3	16.1	21.7	84.8	80.4	74.0
DPPE (C16:0)-PEG$_{2k}$	103.2	85.0	59.0	13.1	28.5	42.5	81.2	65.4	52.0
DSPE (C18:0)-PEG$_{0.55k}$	211.9	156.4	118.7	11.3	10.1	13.3	84.1	82.5	81.3
DSPE (C18:0)-PEG$_{1k}$	156.7	103.2	87.2	11.8	15.8	26.9	83.9	82.6	72.2
DSPE (C18:0)-PEG$_{2k}$	98.4	63.9	77.4	13.8	23.0	57.1	83.7	70.3	62.2
DSPE (C18:0)-2 arm-PEG$_{2k}$	107.2	70.5	52.1	12.9	28.7	49.6	84.8	70.9	52.9
DOPE (C18:1)-PEG$_{0.55k}$	212.1	153.6	120.9	7.1	14.1	15.0	82.8	80.9	78.6
DOPE (C18:1)-PEG$_{1k}$	132.2	100.5	92.9	13.3	17.8	26.3	84.1	78.0	69.7
DOPE (C18:1)-PEG$_{2k}$	104.6	82.2	66.5	13.9	23.9	32.1	83.5	73.2	59.6
DMG (C14:0)-PEG$_{2k}$	116.9	109.6	111.7	11.4	19.0	21.3	80.4	73.6	59.7
DSG (C18:0)-PEG$_{2k}$	114.7	97.3	86.6	11.7	19.4	42.8	80.5	69.9	56.9
Ceramide (C8)-PEG$_{0.75k}$	147.7	118.6	118.9	13.8	12.0	13.9	80.1	78.5	74.8
Ceramide (C8)-PEG$_{2k}$	114.7	116.5	111.9	12.7	16.5	15.2	81.5	67.8	63.4
Ceramide (C16)-PEG$_{0.75k}$	195.8	163.2	130.4	10.1	11.5	23.6	86.2	84.3	83.1
Ceramide (C16)-PEG$_{2k}$	115.6	96.5	96.6	14.3	21.2	22.3	85.0	74.6	53.1

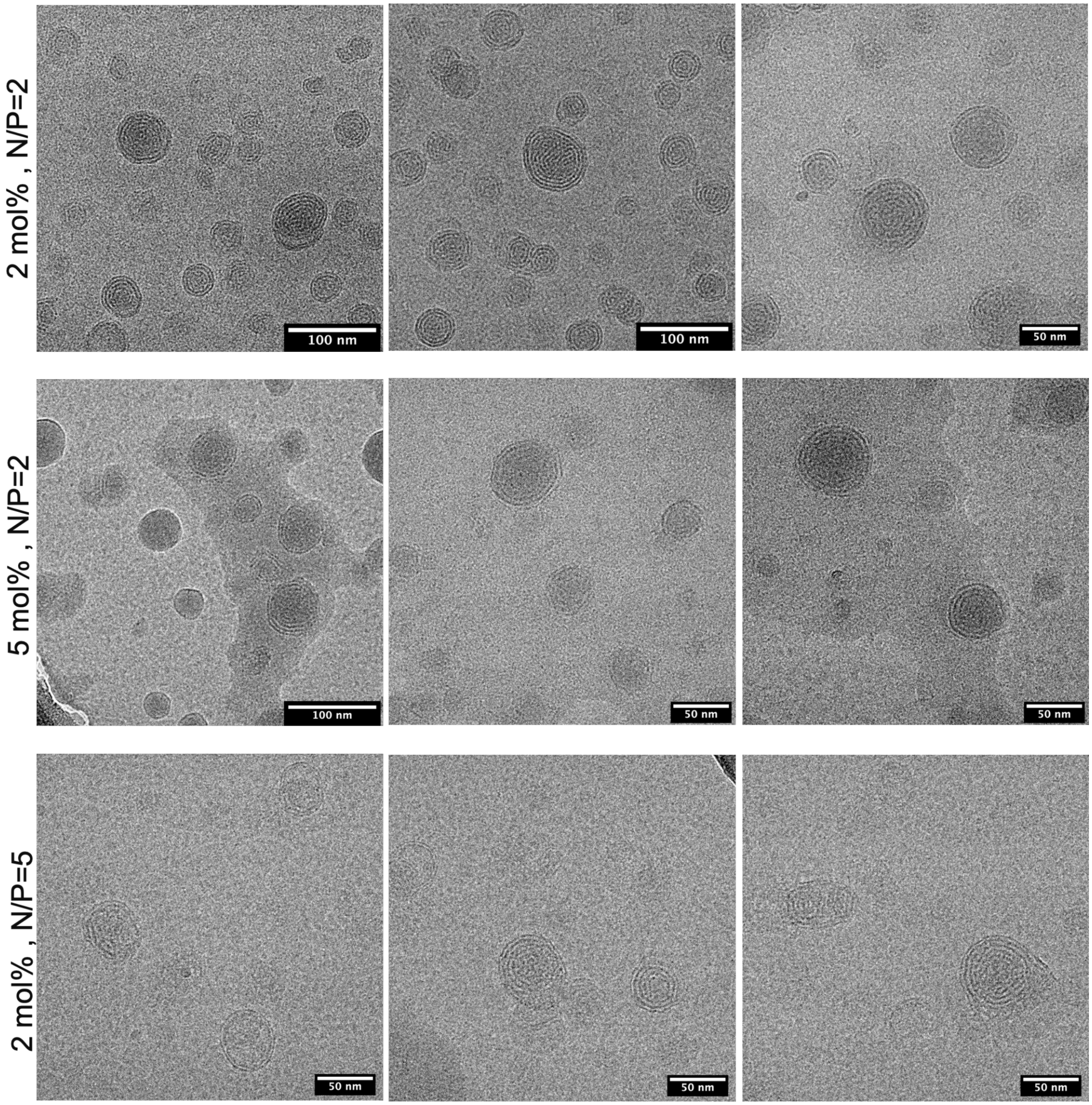

Figure S1. More cryo-EM imaging fields associated with **Figure 1D** support the presence of lamellar and hexagonal phases within pre-purified ASO-LNPs. Molar ratios of the DMG-C14 PEG_{2k} and N/P ratios are indicated.

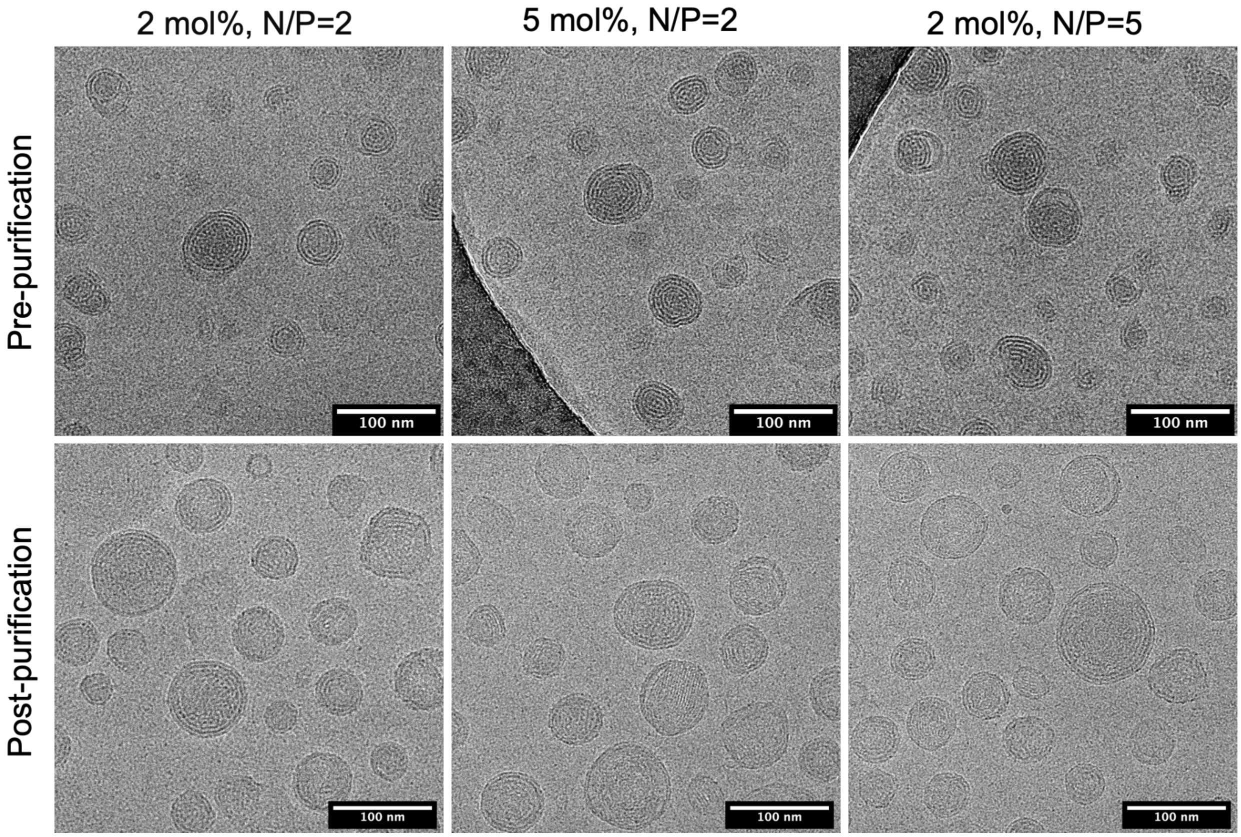

Figure S2. Corresponding cryo-EM images were collected for LNP formulations in pre- and post-purified conditions, as shown in **Figure 1B**. Molar ratios of the DMG-C14 PEG_{2k} and N/P ratios are indicated.

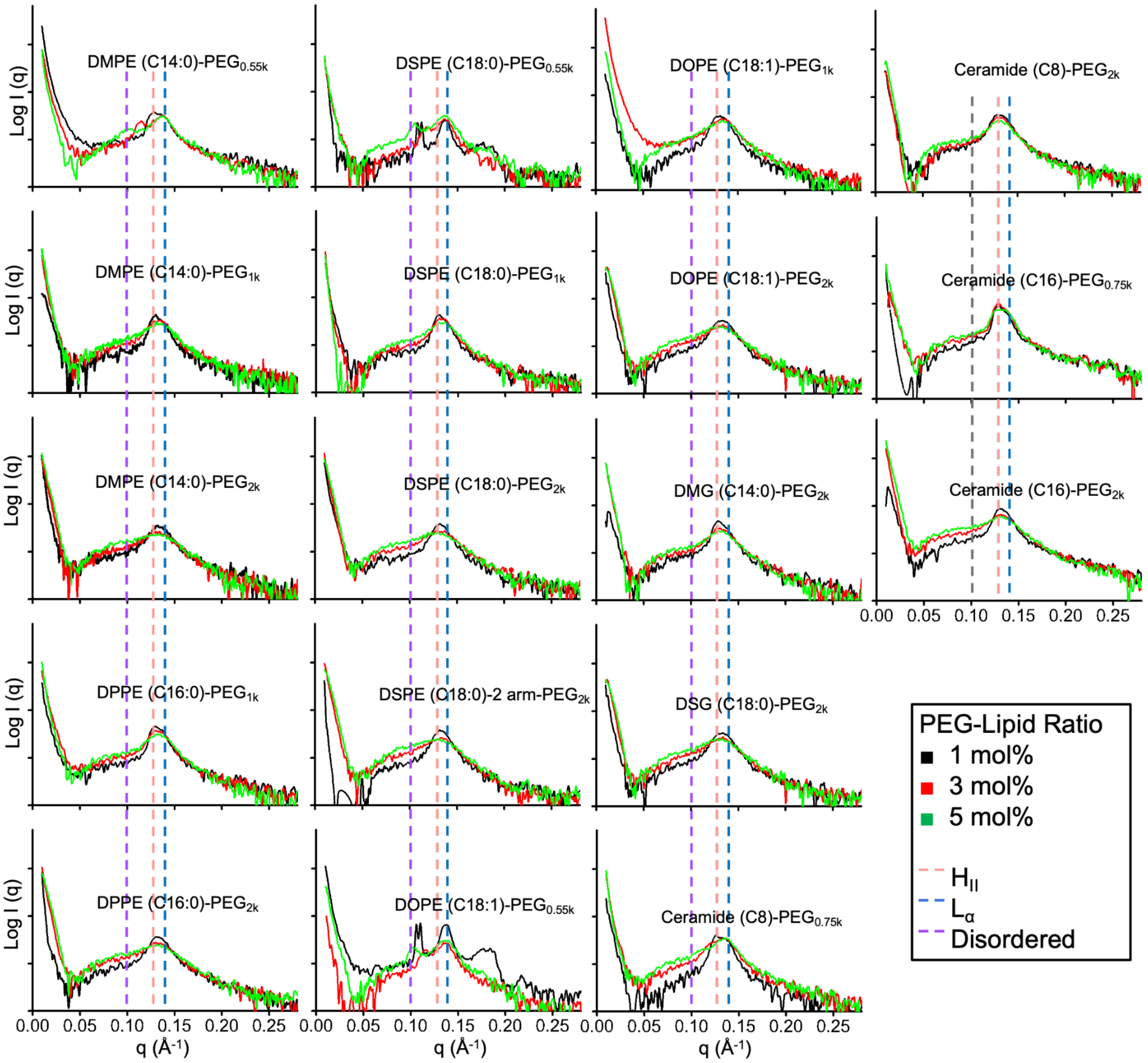

Figure S3. SAXS data across the library of 54 LNP formulations with different PEG-lipids. The positions of the SAXS signature of the disordered, H_{II}, and L_α phases, with the approximate *d* spacing of ~63 Å, ~50 Å, and ~45 Å, are highlighted with violet, red, and blue dashed lines, respectively.

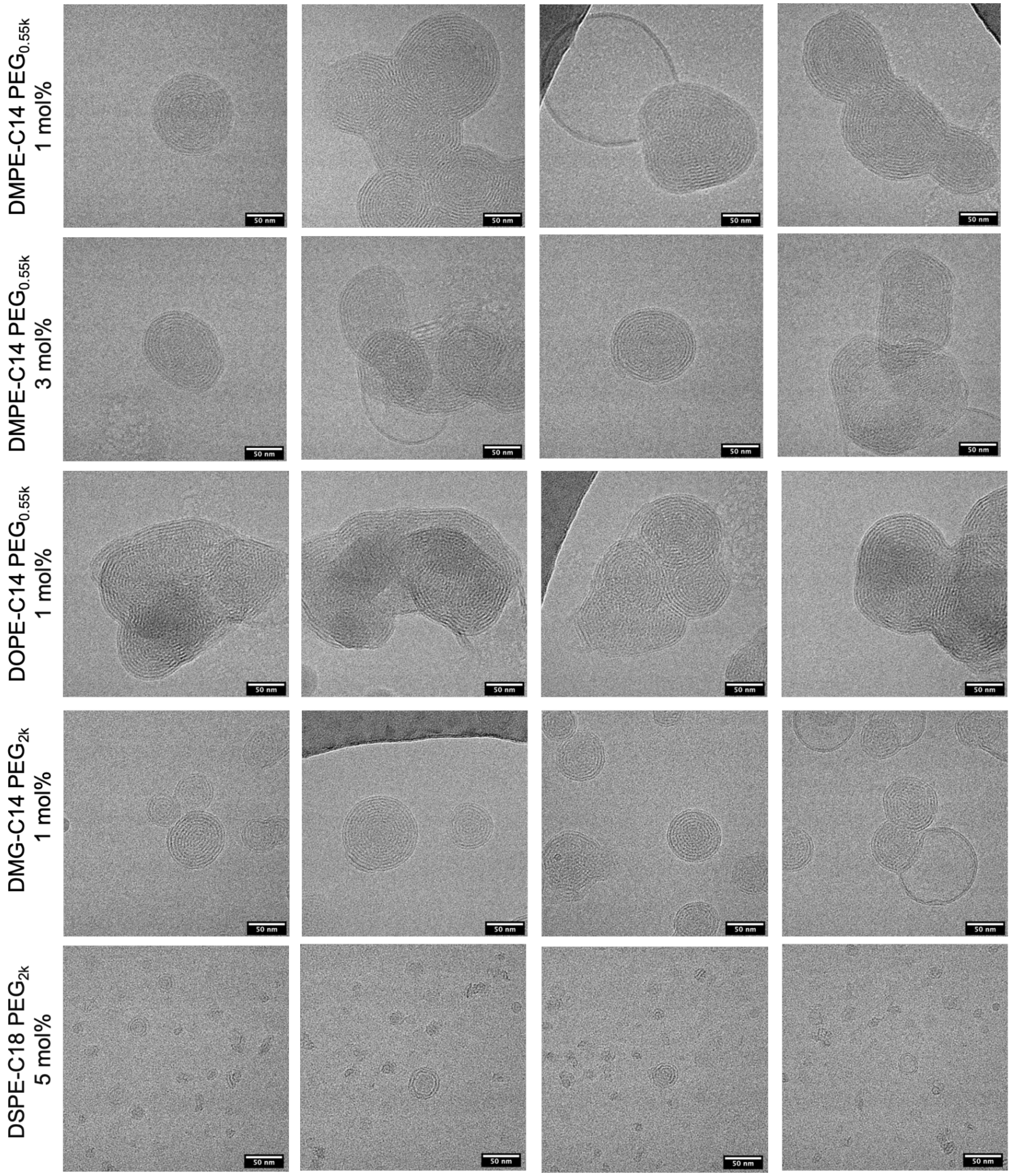

Figure S4. Larger field cryo-EM images associated with **Figure 2B** show variations in the size and morphology of ASO-LNPs with various PEG-lipid compositions.

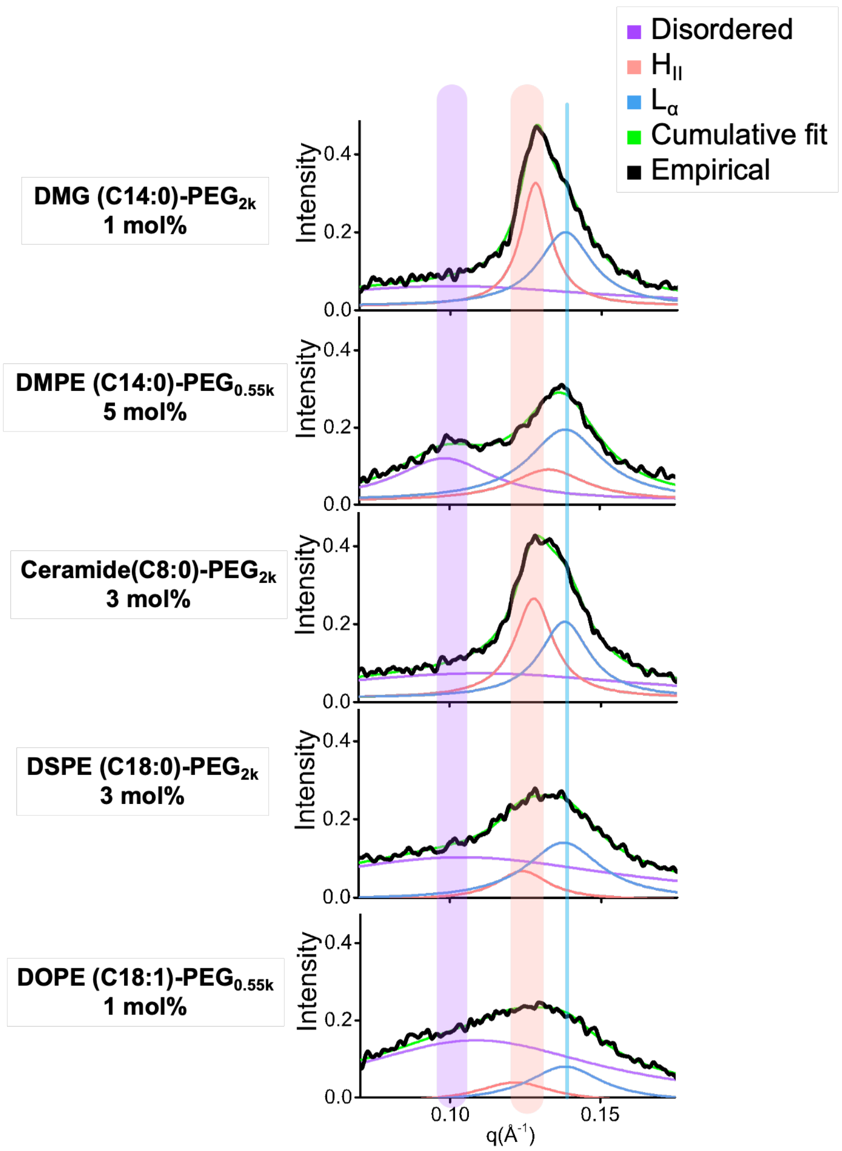

Figure S5. Deconvolution of SAXS peaks of representative LNP formulations. Deconvolution of primary SAXS peaks with three Lorentz functions associated with the disordered, H_{II}, and L_α phases. The center of the Lorentz function and its lower and upper bounds used in the fitting approach are highlighted.

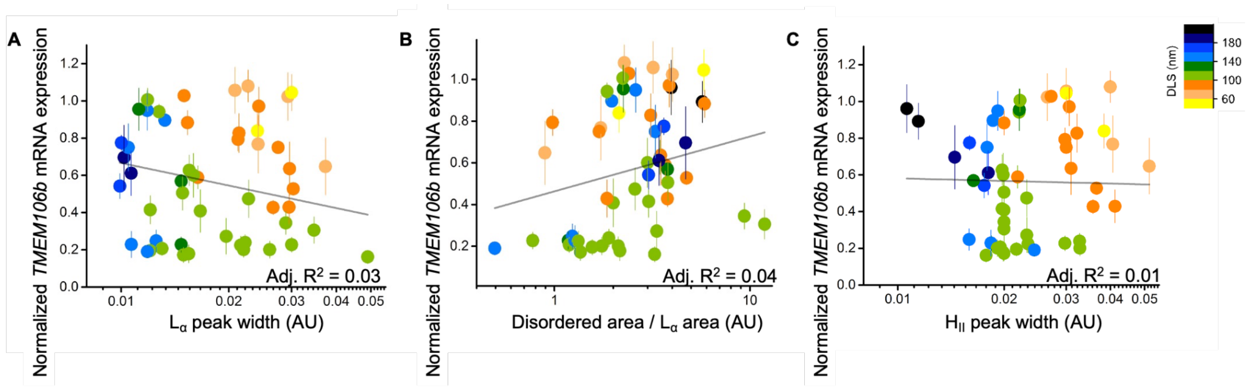

Figure S6. Correlations between additional SAXS peak measures, including the Lorentz function width of the L_α signal (**A**), the ratios of Lorentz function areas representing disordered and L_α phases (**B**), and the Lorentz function width of the H_{II} signal (**C**), with gene knockdown efficacy across the library of 54 LNP formulations.
